# Are antifibrinolytic drugs equivalent in reducing blood loss and transfusion in cardiac surgery? A meta-analysis of randomized head-to-head trials

**DOI:** 10.1186/1471-2261-5-19

**Published:** 2005-07-04

**Authors:** Paul A Carless, Annette J Moxey, Barrie J Stokes, David A Henry

**Affiliations:** 1 Discipline of Clinical Pharmacology, School of Medical Practice and Population Health, Faculty of Health, University of Newcastle, New South Wales, Australia

## Abstract

**Background:**

Aprotinin has been shown to be effective in reducing peri-operative blood loss and the need for re-operation due to continued bleeding in cardiac surgery. The lysine analogues tranexamic acid (TXA) and epsilon aminocaproic acid (EACA) are cheaper, but it is not known if they are as effective as aprotinin.

**Methods:**

Studies were identified by searching electronic databases and bibliographies of published articles. Data from head-to-head trials were pooled using a conventional (Cochrane) meta-analytic approach and a Bayesian approach which estimated the posterior probability of TXA and EACA being equivalent to aprotinin; we used as a non-inferiority boundary a 20% increase in the rates of transfusion or re-operation because of bleeding.

**Results:**

Peri-operative blood loss was significantly greater with TXA and EACA than with aprotinin: weighted mean differences were 106 mls (95% CI 37 to 227 mls) and 185 mls (95% CI 134 to 235 mls) respectively. The pooled relative risks (RR) of receiving an allogeneic red blood cell (RBC) transfusion with TXA and EACA, compared with aprotinin, were 1.08 (95% CI 0.88 to 1.32) and 1.14 (95% CI 0.84 to 1.55) respectively. The equivalent Bayesian posterior mean relative risks were 1.15 (95% Bayesian Credible Interval [BCI] 0.90 to 1.68) and 1.21 (95% BCI 0.79 to 1.82) respectively. For transfusion, using a 20% non-inferiority boundary, the posterior probabilities of TXA and EACA being non-inferior to aprotinin were 0.82 and 0.76 respectively. For re-operation the Cochrane RR for TXA vs. aprotinin was 0.98 (95% CI 0.51 to 1.88), compared with a posterior mean Bayesian RR of 0.63 (95% BCI 0.16 to 1.46). The posterior probability of TXA being non-inferior to aprotinin was 0.92, but this was sensitive to the inclusion of one small trial.

**Conclusion:**

The available data are conflicting regarding the equivalence of lysine analogues and aprotinin in reducing peri-operative bleeding, transfusion and the need for re-operation. Decisions are sensitive to the choice of clinical outcome and non-inferiority boundary. The data are an uncertain basis for replacing aprotinin with the cheaper lysine analogues in clinical practice. Progress has been hampered by small trials and failure to study clinically relevant outcomes.

## Background

Excessive peri-operative bleeding during cardiac surgery involving cardiopulmonary bypass contributes to overall morbidity and mortality [1-6]. Blood loss frequently leads to transfusion of allogeneic blood products, which expose patients to the risk of transfusion-related adverse effects such as febrile non-hemolytic transfusion reactions, transfusion errors and blood-borne infections [2,7]. Concerns about blood safety, continual blood shortages and rising costs of blood bank operations have generated interest in the reduction of transfusion requirements during and after surgery. A popular approach is to minimize peri-operative bleeding through the prophylactic use of the antifibrinolytic agents aprotinin, tranexamic acid (TXA), and epsilon aminocaproic acid (EACA) [8].

Aprotinin, the benchmark compound, is the most widely used and best established antifibrinolytic medication. It is a non-specific broad-spectrum serine protease inhibitor mainly derived from bovine lungs [9]. TXA and EACA are synthetic lysine analogues, which act principally by blocking lysine binding sites on plasminogen molecules, inhibiting plasmin formation and thereby fibrinolysis [10].

Several published systematic reviews have shown aprotinin to be efficacious in reducing peri-operative blood loss, patient exposure to allogeneic blood transfusion and the need for re-operation due to continued or recurrent bleeding [1,2,11-13]. TXA and EACA also have demonstrated efficacy in placebo-controlled trials [1,2,12,13], but the available literature does not allow conclusions to be drawn about the comparative clinical performance of these agents. It is important to establish the relative performance of these agents as aprotinin is substantially more expensive than either TXA or EACA.

In synthesizing the available literature we were interested in whether TXA and EACA are as effective (i.e. no worse than) as the more expensive drug, aprotinin. To achieve this aim we performed a meta-analysis of data obtained from head-to-head randomized controlled trials of aprotinin, TXA, and EACA and performed tests of equivalence (non-inferiority) using a Bayesian approach.

## Methods

### Search strategy

This systematic review was undertaken using the methods established by the Cochrane Collaboration [14]. Databases searched were: Medline (1966–September 2003), EMBASE (1980–September 2003), Current Contents (1993–Week 34 2003) and the Cochrane Central Register of Controlled Trials (CENTRAL – The Cochrane Library, Issue 2, 2003). Initially we used unrestricted search strategies, employing exploded MeSH (Medical Subject Headings) terms and specific text-word terms for aprotinin, tranexamic acid, and epsilon aminocaproic acid. The specific text-word terms included: 'aprotinin', 'antilysin', 'contrical', 'kallikrein-trypsin', 'kallikrein inhibitor$', 'kallikrein inactivator$', 'tranexamic acid', 'cyklokapron', 't-amcha', 'amca', 'amcha', urugol', 'transamin', 'kabi', 'exacyl', 'anvitoff', 'epsilon aminocaproic acid', 'amicar', and 'lederle'. The truncation character "$" was used in Medline and EMBASE to retrieve all possible suffix variations of the root word or phrase. In Medline, EMBASE (Excerpta Medica Database), and Current Contents two search filters were used to restrict and improve the specificity of the electronic database searches. Firstly, the ISPOT (International Study of Peri-operative Transfusion) filter [11] which identifies blood transfusion trials, and secondly, a modified version of the Cochrane Collaboration filter [15], which identifies randomized controlled trials. These search filters were combined with the MeSH and relevant text-word terms for aprotinin, TXA, and EACA. Experts in the field were contacted to identify relevant reports or projects in progress relevant to the review. The bibliographies of identified trials, review articles, and reports were searched for potentially relevant studies. Studies were retrieved regardless of language.

### Study selection criteria

Two reviewers (PAC and AJM) independently evaluated identified articles for eligibility. Studies were eligible for inclusion if they were randomized parallel-group trials, evaluated the drugs as prophylactic interventions in the context of adult elective cardiac surgery, involved the intravenous administration of the trial agents during the pre and/or intra-operative period, and included in their study outcomes the numbers of individuals who received allogeneic RBC transfusions, or the volume of allogeneic RBCs received by subjects in the intervention groups. Duplicate publications, studies involving only children (less than 18 years), and trials that only administered the study drugs during the post-operative period were not considered for review.

### Data extraction

The outcomes measured included: the numbers of patients exposed to allogeneic red blood cell transfusion, and/or the amounts of allogeneic RBC transfused (expressed as units), blood loss (expressed as milliliters), the rates of re-operation for bleeding (re-exploration), non-fatal myocardial infarction, stroke, thrombosis, and mortality. Data were extracted from each trial by two reviewers (PAC and AJM), checked for consistency and accuracy, and then entered into a computer database for analysis.

### Data analysis

Dichotomous data (e.g. required re-operation for bleeding or numbers of patients who were transfused) and continuous data (e.g. mean volume of blood loss and mean units of allogeneic RBC transfused) were analyzed using Cochrane Review Manager 4.1 (MetaView 4.1) [16]. Trials were excluded from analysis if they did not report conventional measures of dispersion (standard deviations or standard errors) along with means for continuous data (or if we were unable to calculate these from the raw data). Data expressed in milliliters (mls) of blood transfused were converted to units by dividing by 300. Outcomes are expressed as pooled relative risks (RR) or weighted mean differences (WMD) (for continuous variables) using a random effects model [17]. The *Q *statistic was used to assess heterogeneity of treatment effect [17]. We also used a Bayesian approach (utilizing WinBUGS software) to model the results of the individual trials as a binomial experiment. We employed a random effects model to calculate the pooled risk ratio, using the methods described by Warn *et al*.[18]. We used a Uniform (0,1) prior for the risk of allogeneic RBC transfusion with aprotinin treatment (consistent with the reported 50% transfusion rate in cardiac surgery) and estimated a prior for re-operation rates with aprotinin from the results of a published systematic review [12]. We integrated the posterior distribution curve for the RR between various pairs of limits to summarize the probabilities of interest. In doing this we were indifferent to the probability of superiority of lysine analogues over aprotinin, but included those areas of the curves in the calculation of the probabilities of non-inferiority. We selected a non-inferiority boundary of 20% (delta value) for re-operation data and the rate of transfusion with allogeneic blood (i.e. TXA & EACA were considered non-inferior to aprotinin if the upper limit of the 95% CI for the pooled RR was ≤1.2). The delta value was varied during sensitivity analysis (i.e. 5% to 40%).

### Assessment of study methodological quality

Studies were assessed for methodological quality by two independent raters (PAC and AJM), using criteria proposed by Schulz *et al*.[19]. These specify four items of assessment: double-blinding, allocation concealment, participant inclusion/exclusion and methods used to achieve randomization. Disagreements were resolved by consensus. Inter-rater agreement for each item of methodological quality was assessed by comparing the observed agreement with that expected by chance. A kappa statistic (which expresses the agreement beyond chance as a proportion of the maximum possible agreement) was calculated for each item assessed. Kappa is equal to one when there is perfect agreement between raters.

## Results

We identified twenty randomized, head-to-head trials involving comparisons of aprotinin TXA and EACA in elective adult cardiac surgery, which reported information on the main outcomes of interest [6,20-38]. One trial [39] was excluded from the analysis due to a lack of usable data (i.e. for continuous data the results were reported as medians [25th–75th percentiles]; the number of patients transfused ≥1 unit of allogeneic RBC transfusion was not reported).

### Characteristics of included studies

The 20 included trials (Tables 1 &2) randomized a total of 2430 subjects to receive either aprotinin, TXA, or EACA. The majority (n = 11) compared aprotinin to TXA. There were only three head-to-head trials of aprotinin versus EACA, three trials of TXA versus EACA, and three trials that compared the three antifibrinolytic drugs with each other. The median size of trial arms was 25 participants (range; 14–522). For each of the three intervention groups the mean age of study participants ranged from 60.5–62.4 years. Most study participants were male (77–79%). The publication period of the trials spanned nine years (1993 to 2001). Only one trial was published in a language other than English and was translated before being included in the analysis [23]. The trials were heterogeneous in terms of drug dose and treatment regimen (Table 3).

**Table 1:** Characteristics of Included Studies

Study	Year	Country	Type of cardiac surgery	Interventions
Isetta *et al*. [25]	1993	France	NR	HD APR (n = 70) vs. LD APR (n = 70) vs. TXA (n = 70) vs. Control (n = 70)
Blauhut *et al*. [27]	1994	Switzerland	CABG	HD APR (n = 14) vs. TXA (n = 14) vs. Control (n = 14)
Penta de Peppo *et al*. [20]	1995	Italy	CABG & Valve Sx.	HD APR (n = 15) vs. TXA (n = 15) vs. EACA (n = 15) vs. Control (n = 15)
Corbeau *et al*. [23]	1995	France	CABG & Valve Sx.	HD APR (n = 43) vs. TXA (n = 41) vs. Control (n = 20)
Pugh *et al*. [22]	1995	UK	Primary CABG	LD APR (n = 21) vs. TXA (n = 22) vs. Control (n = 23)
Speekenbrink *et al*. [21]	1995	The Netherlands	Primary CABG	PP APR (n = 15) vs. TXA (n = 15) vs. DIP (n = 12) vs. Control (n = 15)
Menichetti *et al*. [24]	1996	Italy	Primary CABG	HD APR (n = 24) vs. TXA (n = 24) vs. EACA (n = 24) vs. Control (n = 24)
Pinosky *et al*. [33]	1997	USA	Primary CABG	TXA (n = 20) vs. EACA (n = 20) vs. Placebo (n = 19)
Mongan *et al*. [31]	1998	USA	Primary CABG	HD APR (n = 75) vs. TXA (n = 75)
Hardy *et al*. [26]	1998	Canada	Primary CABG	TXA (n = 42) vs. EACA (n = 46) vs. Placebo (n = 44)
Eberle *et al*. [29]	1998	Germany	Primary CABG	HD APR (n = 20) vs. EACA (n = 20)
Misfeld *et al*. [30]	1998	Germany	Primary CABG	LD APR (n = 14) vs. TXA (n = 14) vs. Control (n = 14)
Casati *et al*. [28]	1999	Italy	Primary CABG & Valve Sx.	HD APR (n = 67) vs. TXA (n = 70) vs. EACA (n = 66)
Bernet *et al*. [34]	1999	Switzerland	Primary CABG	HD APR (n = 28) vs. TXA (n = 28)
Nuttall *et al*. [32]	2000	USA	Re-do CABG & Valve Sx.	HD APR (n = 40) vs. TXA (n = 45) vs. TXA+ANH (n = 32) vs Placebo (n = 43)
Maineri *et al*. [38]	2000	Italy	Primary CABG	TXA (n = 24) vs. EACA (n = 24)
Wong *et al*. [37]	2000	Canada	Re-do CABG & Valve Sx.	HD APR (n = 39) vs. TXA (n = 38)
Casati *et al*. [35]	2000	Italy	Primary CABG & Valve & ASD Repair	HD APR (n = 518) vs. TXA (n = 522)
Greilich *et al*. [36]	2001	USA	Primary CABG	HD APR (n = 24) vs. EACA (n = 23) vs. Placebo (n = 25)
Ray *et al*. [6]	2001	Australia	CABG & Valve Sx.	LD APR (n = 49) vs. EACA (n = 51)

ANH = acute normovolemic hemodilution, APR = aprotinin, ASD = atrial septal defect, CABG = coronary artery bypass graft, DIP = dipyridamole, EACA = epsilon aminocaproic acid, HD = high dose, LD = low dose, NR = not reported, PP = pump prime, Sx. = surgery, TXA = tranexamic acid

**Table 2:** Characteristics of Included Studies

Study	Co-interventions	Transfusion threshold	Anti-platelet use
Isetta *et al*. [25]	PO CS - re-transfusion of SMB	Hct<20% during CPB Hct<25% 4 hrs post CPB Hct<27% post-op.	NR
Blauhut *et al*. [27]	NR	Hct<30% post-op.	Excluded pts. pre-operatively treated with ASA + NSAIDs
Penta de Peppo *et al*. [20]	IO CS + IO & PO re-transfusion of SMB	Post-op. non-monitored pts. Hb<7.0 g/dL Monitored pts. Hb<8.5 g/dL	Discontinued NSAIDs 24 hrs before Sx.
Corbeau *et al*. [23]	NR	Hct<20% during CPB Hct<25% at the end of surgery Hct<30 post extubation	Anti-platelet aggregation drugs ceased 10 days pre-operatively
Pugh *et al*. [22]	IO CS + ANH (1 unit of WB collected pre-CPB then re-transfused post CPB)	Hct<20% during CPB Hct<30% off CPB	Aspirin use within 10 days of the operation: LD APR = 67%, TXA = 91%, Control = 78%
Speekenbrink *et al*. [21]	NR	NR	Aspirin discontinued 2–4 days before Sx.
Menichetti *et al*. [24]	NR	Hct<30% post-operatively	Excluded pts. who had taken ASA or DIP until 2 weeks pre-op.
Pinosky *et al*. [33]	NR	Hct<20% + surgeon preference	Pre-operative aspirin use: TXA = 25%, EACA = 40%, Placebo = 42%
Mongan *et al*. [31]	NR	Hb<6.0 g/dL during CPB Hb<8.0 g/dL off CPB	Pre-operative aspirin use: HD APR = 44%, TXA = 53%
Hardy *et al*. [26]	IO CS & Re-infusion of SMB were not used	Hb<7.0 g/dL during CPB Hb<8.0 g/dL off CPB	NR
Eberle *et al*. [29]	IO & PO CS used	Hct<27% - post-operative + accompanied by signs & symptoms of hypovolemia	Intra-operative IV ASA: HD APR = 5.0%, EACA = 15%
Misfeld *et al*. [30]	NR	Hb<8.0 g/dL	Excluded pts. receiving ASA treatment within 5 days of Sx.
Casati *et al*. [28]	IO CS used + PAD	Hb<6.0 g/dL during CPB Hb<8.0 g/dL off CPB + clinical condition	Pts. receiving ASA treatment within 5 days of Sx.: HD APR = 37.8%, TXA = 40.9%, EACA = 35.3%
Bernet *et al*. [34]	PO CS	Hct<25% PO	All pts. were treated with 100 mg ASA daily until Sx.
Nuttall *et al*. [32]	PAD not used	Hb<7.0 g/dL during CPB	Excluded pts. taking ASA daily (≥325 mg) before Sx.
Maineri *et al*. [38]	IO CS + PO re-infusion of SMB	Hct<30% IO Hct<28% PO	NR
Wong *et al*. [37]	IO CS + PO re-infusion of SMB	Hb<7.0 g/dL IO Hb<8.0 g/dL PO	Excluded pts. receiving ASA treatment within 5 days of Sx.
Casati *et al*. [35]	IO CS used	Hb<6.0 g/dL during CPB Hb<8.0 g/dL PO	Pts. receiving ASA treatment before Sx.: HD APR = 17.8%, TXA = 18.8%
Greilich *et al*. [36]	IO CS used PO SMB was not used	Hb<8.0 g/dL	Pts. receiving ASA treatment before Sx.: HD APR = 88%, EACA = 90%, Placebo = 79%
Ray *et al*. [6]	NR	NR	ASA within 10 days before Sx.: LD APR = 22.4%, EACA = 33.3%

ANH = acute normovolemic hemodilution, APR = aprotinin, ASA = acetylsalicylic acid, CABG = coronary artery bypass graft, CPB = cardiopulmonary bypass, CS = cell salvage, DIP = dipyridamole, EACA = epsilon aminocaproic acid, Hb = hemoglobin, Hct = hematocrit, HD = high dose, LD = low dose, NR = not reported, NSAIDs = non-steroidal anti-inflammatory drugs, PP = pump prime, IO = intra-operative, PO = post-operative, SMB = shed mediastinal blood, Sx. = surgery, TXA = tranexamic acid, WB = whole blood

**Table 3:** Summary of drug dose and treatment regimens

Study	Aprotinin	TXA	EACA
Isetta *et al*. [25]	L = 2.0 × 10^6 ^KIU M = 0.5 × 10^6 ^KIU/h P = 2.0 × 10^6 ^KIU	L = 15 mg/kg	NS
	L = 0.5 × 10^6^ M = 0.5 × 10^6^		
Blauhut *et al*. [27]	L = 2.0 × 10^6 ^KIU M = 0.5 × 10^6 ^KIU/h P = 1.0 × 10^6 ^KIU	L = 10 mg/kg M = 1.0 mg/kg/h	NS
Penta de Peppo *et al*. [20]	L = 2.0 × 10^6 ^KIU M = 0.5 × 10^6 ^KIU/h P = 2.0 × 10^6 ^KIU	L = 10 mg/kg M = 1.0 mg/kg/h	L = 10 g M = 2.0 g/h for 5 h
Corbeau *et al*. [23]	L = 2.0 × 10^6 ^KIU M = 0.5 × 10^6 ^KIU/h P = 2.0 × 10^6 ^KIU	L = 15 mg/kg E = 15 mg/kg	NS
Pugh *et al*. [22]	L = 1.0 × 10^6 ^KIU P = 1.0 × 10^6 ^KIU	L = 2.5 g P = 2.5 g	NS
Speekenbrink *et al*. [21]	P = 2.0 × 10^6 ^KIU	L = 10 mg/kg M = 1.0 mg/kg/h	NS
Menichetti *et al*. [24]	L = 2.0 × 10^6 ^KIU M = 0.5 × 10^6 ^KIU/h P = 2.0 × 10^6 ^KIU	L = 10 mg/kg M = 3.0 mg/kg/h P = 10 mg/kg	L = 80 mg M= 30 mg/kg/h P = 80 mg/kg
Pinosky *et al*. [33]	NS	L = 15 mg/kg M = 1.0 mg/kg/h for 6 h	L = 150 mg/kg M = 10 mg/kg/h for 6 h
Mongan *et al*. [31]	L = 2.0 x 10^6 ^KIU M = 0.5 × 10^6 ^KIU/h P = 2.0 × 10^6 ^KIU	L = 15 mg/kg M = 2.0 mg/kg/h for 6 h	NS
Hardy *et al*. [26]	NS	L = 10 g	L = 15 g M = 1.0 g/h
Eberl *et al*. [29]	L = 2.0 × 10^6 ^KIU M = 0.5 × 10^6 ^KIU/h P = 2.0 × 10^6 ^KIU	NS	L = 10 g M = 2.5 g/h P = 10 g
Misfeld *et al*. [30]	P = 1.0 × 10^6 ^KIU E = 0.2 × 10^6 ^KIU/h for 5 h	L = 10 mg/kg M = 1.0 mg/kg/h	NS
Casati *et al*. [28]	L = 2.0 × 10^6 ^KIU M = 0.5 × 10^6 ^KIU/h P = 2.0 × 10^6 ^KIU	L = 1.0 g M = 400 mg/h P = 500 mg	L = 5.0 g M = 2.0 g/h P = 2.5 g
Bernet *et al*. [34]	L = 2.0 × 10^6 ^KIU M = 0.5 × 10^6 ^KIU/h P = 2.0 × 10^6 ^KIU	L = 10 g	NS
Nuttall *et al*. [32]	L = 2.0 × 10^6 ^KIU M = 0.5 × 10^6 ^KIU/h P = 2.0 × 10^6 ^KIU	L = 10 mg/kg M = 1.0 mg/kg/h	NS
Maineri *et al*. [38]	NS	L = 20 mg/kg M = 2.0 mg/kg/h	L = 10 g M = 2.0 g/h
Wong *et al*. [37]	L = 2.0 × 10^6 ^KIU M = 0.5 × 10^6 ^KIU/h P = 2.0 × 10^6 ^KIU	L = 10 g	NS
Casati *et al*. [35]	L = 2.0 × 10^6 ^KIU M = 0.5 × 10^6 ^KIU/h P = 2.0 × 10^6 ^KIU	L = 1.0 g M = 400 mg/h P = 500 mg	NS
Greilich *et al*. [36]	L = 2.0 × 10^6 ^KIU M = 0.5 × 10^6 ^KIU/h P = 2.0 × 10^6 ^KIU	NS	L = 100 mg/kg M = 2.5 mg/kg/h P = 5.0 g
Ray *et al*. [6]	L = 1.0 × 10^6 ^KIU P = 1.0 × 10^6 ^KIU	NS	L = 5.0 g M = 1.25 g/h P = 5.0 g

L = loading dose, M = maintenance dose/continuous infusion, P = pump prime dose, E = after protamine administration, KIU = kallikrein inhibitor units, NS = not studied, mg = milligram, g = gram, kg = kilogram, h = hour

### Methodological quality of the studies

Nineteen of the 20 trials were assessed for methodological quality by the two raters (PAC and AJM). As the non-English language study [23] could not be adequately assessed by the two raters, it was excluded from the analysis of the reliability of quality assessment procedure. For the four items of the Schulz criteria [19] used to assess trial quality, the observed agreement was good with kappa scores ranging from 0.92 to 1.0. Generally, the methodological quality of the trials reviewed was poor. Double-blinding was reported in eight trials (42%), concealment of treatment allocation was judged to be adequate in four trials (21%), and only four trials (21%) described the method used to generate allocation sequences (i.e. randomization procedure). Follow-up was judged to have been complete in five trials (26%). For seven trials there was incomplete follow-up; however for these trials only a small number of exclusions were reported making differential withdrawal an unlikely source of bias. For the seven remaining trials a rationale for the withdrawal of study subjects was not provided. As the majority of trials were of poor methodological quality stratification of the data by methodological quality and subgroup analyses were uninformative. We were therefore unable to determine whether treatment effect estimates varied due to study methodological quality.

### Meta-analyses

#### TXA vs. Aprotinin (10 trials, 1707 subjects)

**Figure 1:**
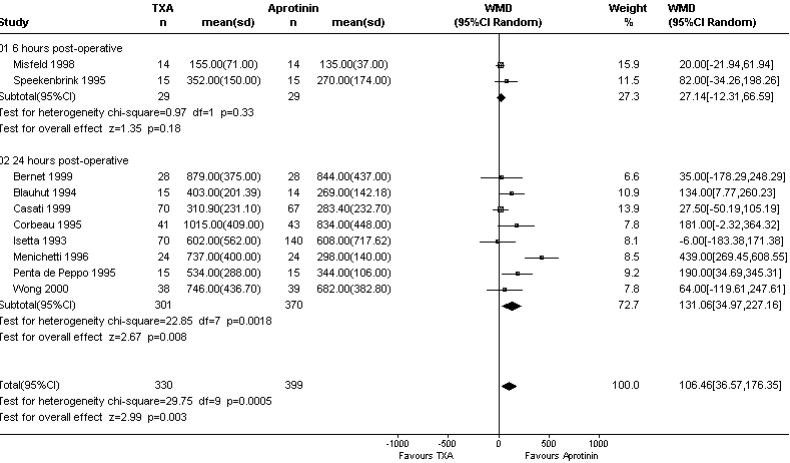
Forest plot of 10 comparative trials of TXA and aprotinin – weighted mean difference in blood loss.

**Figure 2:**
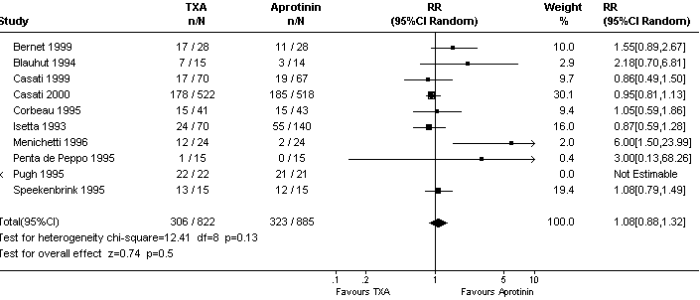
Forest plot of 10 comparative trials of TXA and aprotinin – pooled relative risk of requiring an allogeneic red cell transfusion.

**Figure 3:**
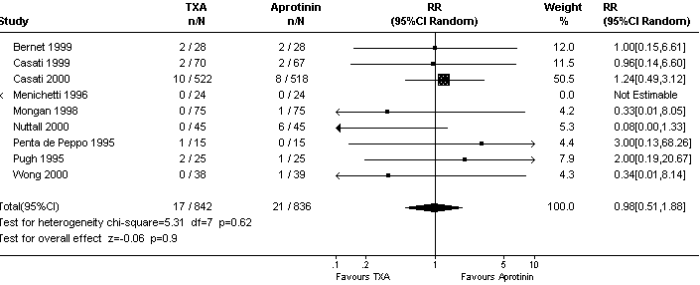
Forest plot of 9 comparative trials of TXA and aprotinin – pooled relative risk of needing re-operation for bleeding.

On average, TXA was inferior to aprotinin in reducing 24 hour blood loss (WMD 106 mls, 95% CI 37 to 176 mls; Fig 1). This apparent disadvantage of TXA was not reflected in the transfusion data. For the five trials (N = 357 subjects) that reported on the amount of blood transfused, the mean numbers of red cell units did not differ between the two drugs; WMD 0.06 units (95% CI -0.18 to 0.31 units). The rate of red cell transfusion in patients treated with TXA was 37.2% compared with 36.5% with aprotinin (Cochrane RR 1.08, 95% CI 0.88 to 1.32; Fig 2). The equivalent Bayesian posterior mean relative risk was 1.11 (95% BCI 0.92 to 1.45). Data on re-operation rates were sparse (Fig 3). The Cochrane estimate of the pooled RR for re-operation with TXA compared to aprotinin was close to one (RR 0.98, 95% CI 0.51 to 1.88). In contrast, the Bayesian posterior mean risk ratio was 0.63 (95% BCI 0.16 to 1.46). Most of the difference between TXA and aprotinin seemed to be contributed by one study (Nuttall *et al*.[32]). This study reported re-operation rates of 0/45 with tranexamic acid and 6/45 with aprotinin, equating to an absolute risk reduction of 13% [risk difference (RD) -0.13, 95% CI -0.24 to -0.03]. In comparison, none of the remaining trials reached statistical significance for this outcome with the risk differences ranging from -0.03 to 0.07 and the 95% confidence intervals including unity (RD = 0). Excluding the data from this one trial [32] changed the mean posterior RR to 0.93 (95% BCI 0.30 to 1.96).

**Figure 4:**
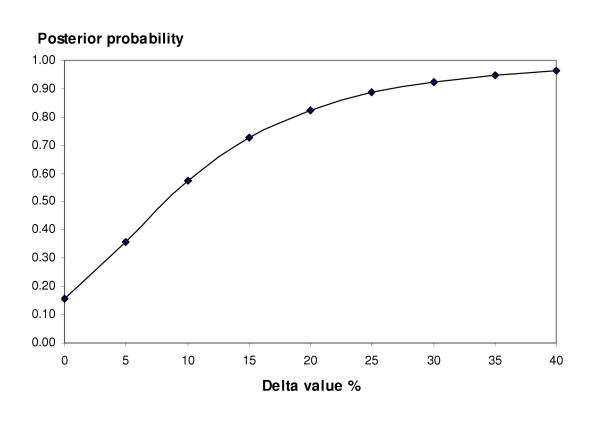
Posterior probability of TXA being considered non-inferior to aprotinin at different delta values (transfusion data).

For RBC transfusion the estimated posterior probability of non-inferiority TXA to aprotinin (with a pooled RR threshold of 1.2) was 0.82. If the threshold was set to 1.1 the posterior probability of non-inferiority was 0.57 (Fig 4). The probabilities of non-inferiority of TXA for re-operation were higher than for transfusion, being 0.92 and 0.90 for the delta values of 20% and 10% respectively, but fell to 0.69 and 0.64 when the data from Nuttall *et al*.[32] were excluded from the calculations.

#### EACA vs. Aprotinin (6 trials; 399 subjects)

**Figure 5:**
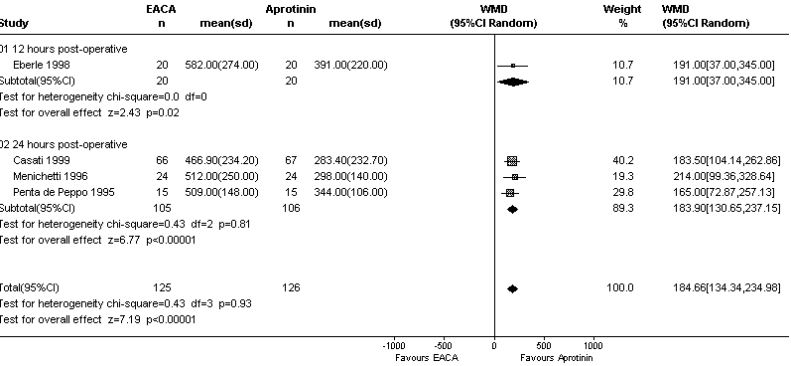
Forest plot of 4 comparative trials of EACA and aprotinin – weighted mean difference in blood loss.

**Figure 6:**
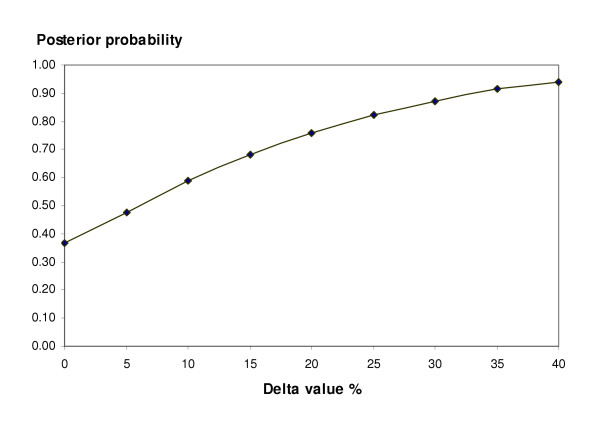
Posterior probability of EACA being considered non-inferior to aprotinin at different delta values (transfusion data).

EACA was inferior to aprotinin in controlling blood loss over 24 hours (WMD 184 mls, 95% CI 134 to 235 mls; Fig 5). However, the mean number of units of allogeneic RBC transfused did not differ between the drugs (WMD -0.22 units, 95% CI -0.52 to 0.09 units). Transfusion rates were similar for EACA and aprotinin: Cochrane RR 1.14 (95% CI 0.84 to 1.55); Bayesian posterior mean risk ratio 1.08 (95% BCI 0.73 to 1.52). Using a non-inferiority threshold value of 1.2 for the pooled RR, the probability of EACA being non-inferior to aprotinin was 0.76. With the threshold set at 1.1 the posterior probability of non-inferiority dropped to 0.54 (Fig 6). There were insufficient data to analyze the effects of treatment on re-operation rates.

#### Other outcomes

Analyses of other clinical outcomes such as all cause mortality, myocardial infarction and stroke were generally uninformative because of the sparse data, but we saw no trends favoring any of the drugs studied here, compared with the others (data not displayed).

Direct comparisons between TXA and EACA revealed no clinically meaningful or significant differences therefore we did not perform non-inferiority tests for these two agents.

## Discussion

Aprotinin has become a widely used adjunct in cardiac surgery [2], a practice that is supported by the results of a large number of placebo-controlled trials [1,11,12]. These trials have demonstrated reductions in allogeneic red cell transfusion, and the need for re-operation due to bleeding. Placebo-controlled trials of tranexamic acid (TXA) and epsilon aminocaproic acid (EACA) have also demonstrated efficacy, but the data are sparse and it is unclear from the published indirect comparisons whether they are as effective as aprotinin [11,12]. This is not an academic question as both agents are substantially cheaper than aprotinin. For example, an average course of treatment with aprotinin in Canada costs CAN$1000, compared with CAN$100-275 for TXA and approximately CAN$50 for EACA [37].

So there are financial pressures to switch from aprotinin to the synthetic lysine analogues. But this should only be contemplated if there is a high degree of confidence that the treatments are clinically equivalent. Conventional meta-analysis provides pooled estimates of differences between treatments (with uncertainty reflected in the width of the confidence intervals). But to demonstrate an acceptable level of 'equivalence' we need to estimate and interpret the probability of a drug's efficacy lying within a 'non-inferiority' boundary [40]. We have to make a judgment about what level of non-inferiority is acceptable, and agree on a tolerable probability of breaching this threshold. These are difficult judgments and we accept that our approach is somewhat arbitrary.

In this paper we used the rates of blood loss, transfusion with allogeneic red cells and re-operation due to continued or recurrent bleeding as the outcome variables. Adequate mortality and morbidity data were not available from the trials. Both lysine analogues seemed inferior to aprotinin in controlling peri-operative blood loss, but the increments were small (between 100 and 200 mls), and of uncertain clinical significance. In the case of red cell transfusions we set the non-inferiority boundaries at 1.2 (a relative 20% increase) in the base case analyses. The rate of transfusion for aprotinin-treated patients in these trials was around 35%, therefore a non-inferiority threshold of 1.2 translates into an absolute increase of around 6.9% in transfusion frequency in this population. In the case of TXA the probability of non-inferiority with this threshold was 0.82, but was slightly lower in the case of EACA (0.76) because of sparse data. To achieve a higher level of confidence in the 'equivalence' of TXA, for example 90%, it is necessary to tolerate a non-inferiority boundary of 1.4 – an absolute increase in the transfusion rate of around 12%. It is difficult to know how this will be viewed by clinicians, but some may consider it as an unsatisfactory basis for switching from a drug of proven efficacy.

As blood transfusion is a practice variable, as opposed to a clinical end-point variable, it requires a degree of subjectivity on the part of clinicians. The decision to transfuse is complex and sometimes arbitrary. It will be influenced by local transfusion protocols, the patient's pre-operative hemoglobin (Hb), the estimated degree of blood loss and the presence of co-morbidity (particularly coronary disease). We do not think that such decisions are likely to be sensitive to the modest differences in blood loss reviewed here, in fact that is what the data indicate.

Our analyses encouraged us to have greater confidence in the equivalence of TXA to aprotinin in preventing the need for re-operation than the need for transfusion. But we remain uncertain about these data. For re-operation, with the threshold for the pooled RR set at 1.2, the probability of TXA being non-inferior to aprotinin was 0.92. This is moderately higher than the probability of 0.82 for RBC transfusion. This is because the Bayesian estimate for the posterior mean RR was 0.63, with a high proportion of the posterior probability distribution below a value of 1.0. Consequently, the integrated area below the non-inferiority boundary of 1.2 was high. Re-operation was uncommon in this population, being required by only 2.5% of aprotinin recipients. Although the point estimates of the RR suggested a trend in favor of TXA (not seen for other outcomes), the confidence intervals were wide and the results changed (unfavorably for TXA) when a single small trial (Nuttall *et al*.[32]), which contributed disproportionately to the difference between the drugs, was excluded from calculation. In addition, these trends are not paralleled by improvements in blood loss (which was worse with TXA than with aprotinin) or transfusion requirements. For these reasons we think that the findings should be interpreted cautiously.

Heterogeneity in trial outcomes was not particularly prominent in our analyses. For the main study outcome (i.e. number of patients transfused allogeneic blood) heterogeneity was not statistically significant (TXA vs. aprotinin, p = 0.13; EACA vs. aprotinin p = 0.55). Although the results for blood loss indicated statistically significant heterogeneity (TXA vs. aprotinin, p = 0.0005) it appears that the data from one trial contributed to this result (Menichetti *et al*., 1996). When the data from this trial were removed from the analysis heterogeneity was no longer significant (p = 0.29).

We were unable to formally assess the impact that the use of anti-platelet agents had on treatment effect estimates as the majority of trials either excluded patients that had been treated with acetylsalicyclic acid (ASA) or dipyridamole (DIP) within 5–10 days of surgery or discontinued treatment with these agents pre-operatively to avoid excessive bleeding. However, in those trials that included ASA or DIP treated patients generally treatment with these agents was evenly distributed across trial arms.

Stratification of trial data by the use of cell salvage proved only marginally informative. Subgroup analysis indicated that for the six trials that used cell salvage the pooled relative risk of receiving an allogeneic RBC transfusion in those patients treated with TXA was 0.97 (95%CI 0.84 to 1.12) compared to 1.54 (95%CI 0.82 to 2.91) for the four studies that did not report the use of cell salvage. Although there appeared to be a trend toward a reduced risk of transfusion in those trials that used cell salvage both results failed to reach statistical significance with the 95% confidence intervals crossing unity. For EACA subgroup analysis was uninformative due to the small number of trials.

## Conclusion

The conclusions that can be drawn from these data are limited for a number of other reasons. The studies were of generally poor quality. This is regrettable as trials of drugs are generally easier to conduct well than trials of different transfusion thresholds or surgical techniques. We have not examined the data for publication bias and are uncertain what effect this might have as the trial comparisons involved active treatments. We have not explored heterogeneity in detail, but it was not particularly prominent in these analyses. The main limitation was the small size of the trials and the reliance on transfusion rates rather than more clinically meaningful endpoints. Doubts about the clinical performance of a treatment are tolerable when the clinical consequences are slight. However, when the result of treatment failure is an unplanned visit to the operating theatre and a further sternotomy or thoracotomy to deal with the source of continued bleeding we need assurance about the equivalence of our treatment choices. In our view the data reviewed here do not provide this reassurance and larger comparative studies using clinically important endpoints are necessary.

## Competing interests

The author(s) declare that they have no competing interests.

## Authors' contributions

PAC conducted the literature search, screened articles for eligibility, assessed methodological quality of included studies, extracted data, analyzed data, interpreted results, and wrote manuscript. AJM screened articles for eligibility, assisted with data extraction and methodological assessment of included studies. BJS performed Bayesian analysis of data and provided statistical consultancy for this project. DAH conceived study project and provided critique of successive drafts of the manuscript. All listed authors read and approved the final manuscript.

## Pre-publication history

The pre-publication history for this paper can be accessed here:


